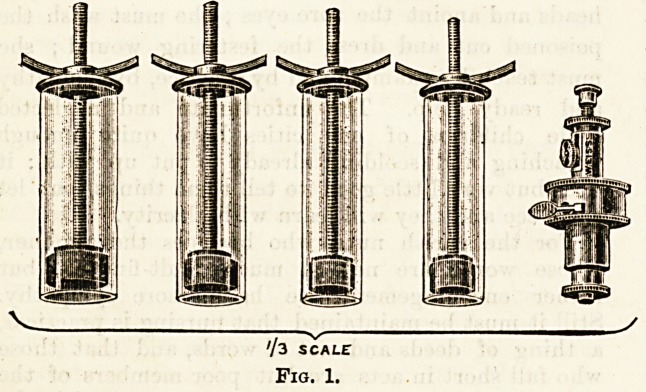# The Hospital. Nursing Section

**Published:** 1903-08-22

**Authors:** 


### The Hospital.

**Nursing Section.**

Contributions for this Section of "The Hospital" should be addressed to the Editor, "The Hospital" Nursing Section, 28 & 29 Southampton Street, Strand, London, W.C.

No. 882.—Vol. XXXIV. *SATURDAY, AUGUST* 22, 1903.

### Notes on News from the Nursing World.

### OUR CHRISTMAS DISTRIBUTION.

It has been our custom in September to remind our readers of our Christmas distribution of useful articles of clothing for the patients in hospitals and infirmaries. We make our first appeal a little sooner this year because the success of our experiment last year in exhibiting the contributions a few days before they were dispatched, still further increased the interest taken in the matter, and suggested that by calling attention a month or so earlier to the matter, we might enlist even wider sympathy and help on this occasion. While the holidays are in full swing, it may be easier for some who desire to assist, to spare time to make the class of garments which are so greatly appreciated, and if they want to get them out of the way they can be sent directly they are ready, addressed to the Editor, 28 and 29 Southampton Street, Strand, London, W.C., with "Clothing Distribution" marked on the outside of the parcel.

### THE MATRON-IN-CHIEF OF THE MILITARY NURSING SERVICE.

In April last year it was officially announced that Miss Sidney Browne had been appointed temporarily to the post of matron-in-chief of Queen Alexandra's Imperial Military Nursing Service. On Saturday last Miss Browne's permanent appointment was gazetted. We are sure that her confirmation in the high and important office for which she has shown herself so remarkably well qualified, will give complete satisfaction, not only to the members of the service itself, but likewise to all who have come in contact with her. At the time the selection was made, we observed that the varied and distinguished career of Miss Browne, who possesses both the South African medal and the decoration of the Royal Red Cross, amply justified the choice. Since she has filled the position, her tact, judgment, and kindly sympathy, have been apparent to every one associated with her; and so long as she is the working head of the Military Nursing Service there will be no danger of discontent in the ranks.

### THE INDIAN NURSING SERVICE.

The revised regulations of the Indian Nursing Service, which is now called Queen Alexandra's Military Nursing Service for India, appear in another page. The alterations include the division of the service into three grades instead of two, there being at present 65 nursing sisters, 15 senior nursing sisters, and four lady superintendents. There is a slight improvement in the pay of the senior nursing sisters, and special rates of gratuity and pension are given for service in that grade. As we intimated some time ago, the service, though its name has been appropriately altered, remains perfectly distinct, and will be governed, as hitherto, by the India Office. A form of the declaration which every candidate is required to make can be obtained on application to the Under Secretary of State, India Office, St. James' Park, S.W.

### A VICTORIAN ARMY NURSING SERVICE.

From Victoria comes the news that it is shortly intended to establish, in connection with the military forces of Victoria, an Army Nursing Service. It is stated that the service will consist, at the outset, of a lady superintendent, a matron, and twenty-five nurses.

### RECOGNITION BY THE ADMIRALTY OF A NURSE'S BRAVERY.

The Lords of the Admiralty have officially recognised the bravery of Miss Chadwick, a nurse at Las Palmas, in the Canary Islands. In February this year a number of natives of Puerto de la Luz savagely assaulted the captain of his Majesty's ship *Isis*, and at a critical moment in the fray Miss Chadwick faced the furious mob and saved the captain from further danger. In a letter dated May 30th, addressed to Miss Knowles, matron of the British Seamen's Hospital, Puerto de la Luz, the Lords of the Admiralty state that, having received from the captain of the *Isis* an account of the circumstances connected with the assault committed on him the previous February, they desired to convey to her and to those under her charge the expression of their sincere thanks for the kindness and attention shown to Captain Munday whilst he was under treatment in the British Seamen's Hospital. Their lordships went on to say that they considered "that the bravery displayed by Nurse Chadwick in facing the mob at a most critical moment, and at her personal risk checking further violence, deserved special recognition at their hands," and they therefore desired the matron to deliver to Miss Chadwick a watch and chain as a mark of their high appreciation of the services she had rendered. The gold watch bears the following inscription:—"Presented to Nurse Chadwick by the Lords Commissioners of the Admiralty for conspicuous service rendered to the captain of H.M.S. *Isis*, 24 February, 1903."

### LORD ROBERTS AND THE ALEXANDRA NURSE.

On the occasion of the military service which the Commander-in-Chief attended at St. Giles's, Edinburgh, the other Sunday, he observed among the crowd waiting to see him as he passed out one of the Alexandra nurses, Miss A. M. Brown—who has recently come home on leave—and at once stopped and spoke most kindly to her, remarking on the

South African medals she wore. Miss Brown was the only nurse belonging to the Soldiers and Sailors' Families' Association in South Africa during the war, and at the time had 580 women and children under her sole charge. Her work was greatly increased at the beginning of hostilities in Ladysmith, when the women and children were hurriedly sent down to her care in Pietermaritzburg. After the majority of them were sent home to England, Nurse Brown rendered valuable service at the Inspection Room, Fort Napier, many thousands of sick and wounded soldiers coming under her care. Twice before this, she has had the honour of being presented to Lord Roberts in Pietermaritzburg, while she was on duty in the capital of Natal.

### BOMBAY NURSES AND THE MUNICIPALITY.

The Bombay Municipality, having refused to undertake to reimburse the nurses engaged at the Arthur Road Hospital the losses which they sustained at a recent fire in their quarters, an appeal for help was promptly made to the public. It is significant that the retiring governor of Bombay, Lord Northcote, at once subscribed a hundred rupees, thus showing in an emphatic manner his sense of the conduct of the Municipality, and 1,667 rupees were soon obtained. There is, happily, no danger that the nurses will be out of pocket by a disaster for which they were in no way responsible, and it is satisfactory to learn that the Hindu and Mahomedan citizens of Bombay joined with British residents in condemning the meanness of the Municipality, and in contributing to the compensation fund.

### NURSES AS BRIDESMAIDS.

With reference to a wedding reported in a local newspaper at West Croydon Tabernacle, at which the bridesmaids were described as being attired in nurses' uniforms, a letter from one of these young ladies and one from the father of the bride appear in another column. The former informs us that the bride and all the bridesmaids were nurses and members of the Royal British Nurses' Association, and expresses an opinion that, this being so, an apology is due from us to both bride and bridesmaids. Mr. Jayne, the father of the bride, also writes to us, in a less temperate strain, and states that he sent us the report of the wedding, which does not mention that any of the ladies were nurses, but says, "the bridesmaids being in nurses' uniforms, blue cornflower dresses, white aprons, caps, gloves, and carrying cornflower bouquets, which was most applicable, the bride having served many years in hospital work." We gladly admit, now we know the facts that there has been no "abuse" of nurses' uniform, in so far as the bridesmaids had a perfect right to wear it. But even so, we think that the uniform of a nurse, which suggests pain and suffering, should not be used by a party of bridesmaids because it is considered a pretty and becoming costume.

### NURSING IN EGYPT.

It is advisable that nurses who intend to reside in Egypt should give attention to the metric system which is used not only in nursing but in all the shops. At present there is no Egyptian pharmacopœa, but one is understood to be in process of compilation. Next to Arabic, French is the most useful language. For the first winter in Cairo the clothing should be similar to that worn in an English autumn or early spring. Woollen under-garments are a necessity, with a medium wrap or coat for evening wear. In summer, muslins and thin materials are best, and during the second winter nurses can dress precisely as they do in the winter in England. The nurse's cloak should be very light, and bonnet strings are not successful, owing to the heat. A linen sailor hat, with wide brim and a band to match the cloak, similar to the motor hats in some of the London shop windows may be found more suitable than a bonnet. As the new Anglo-American Hospital will be opened next winter, some of our readers may be glad of information which is the result of personal experience. It may be added that the hospital is situated on the Island of Ghezireh, which is divided from Cairo by the Kasr-el-Nil Bridge. An electric car runs frequently from Ghezireh to the Pyramids and Sphinx, passing the Zoological Gardens.

### THE ANGLO-AMERICAN NURSING HOME AT ROME.

Further information comes to us respecting the unsatisfactory state of affairs at the Anglo-American Nursing Home in Rome. It is not, we are glad to say, disputed by our informants that the home has been of very great service in the past. The difficulty appears to be that the committee, which consists of members of the Embassy and others in influential social position, who have been extremely successful in getting subscribers, are quite satisfied, and resent any interference, while under present auspices, after the ignored complaints of the entire staff, it cannot be expected that others will readily attach themselves to the institution. It is, of course, exceedingly important that English and American visitors to Rome who may fall ill should be able to command the services of the best nurses, and so far inmates of the Anglo-American Home have enjoyed that advantage. Not only the committee, but the subscribers also, will be to blame if in this respect there is any falling off in the future.

### DERBY NURSES ENTERTAINED BY THE MAYOR.

The entertainment by the Mayor of Derby, Mr. Frederick Strutt, of a number of nurses at Lea Hurst, the former residence of Miss Florence Nightingale, was followed on Thursday, last week, by one of a similar character at Milford House, Duffield, the mayor's own residence, the guests enjoying a most delightful afternoon. Mr. Strutt, in welcoming about 70 nurses connected with the various institutions in Derby and the district, said that he was very glad to meet them, and although they were not at Lea Hurst, the letter which Miss Nightingale wrote to the nurses the previous week was just as much for them. He accordingly proceeded to read it, and then introduced Miss Newton, whom he described as having devoted her life to nursing in the East and Palestine. Miss Newton, in the course of an extremely interesting address, related some of her own experiences nearly a quarter of a century ago, at Jaffa, where with another lady she was mainly instrumental in securing the erection of the now well-known English hospital, containing 40 beds, with a staff consisting of an English and a native doctor, and seven nurses. She also referred to the epidemic of cholera in Jaffa last winter, and to the assistance rendered by a fund, contributed by Derbyshire people, for the relief of the sufferers, and stated that the natives were very grateful for all that was done. The mayor, in proposing a vote of thanks to Miss Newton, observed that the nurse's profession was a noble one, and that although all nurses could not do so much as Miss Nightingale or Miss Newton, they could do their best.

### SEA-GOING NURSES.

The promoters of the movement in favour of appointing trained nurses on board steamers may cite, not only the death of a passenger on an Isle of Man steamer, but the miserable experiences of many of the travellers by the Channel steamers the last few days, in support of the contention that, even in respect to vessels plying merely short distances, action on the part of the steamship companies is needed. Since we drew attention again to the subject, we have had numerous inquiries from nurses anxious to take up such work, and there is not the least excuse for the companies continuing to provide only untrained ex-domestic servants to attend to the sufferers from sea-sickness under their care.

### ABOLITION OF PROBATIONERS AT CHRISTCHURCH UNION INFIRMARY.

The Christchurch Guardians have decided to do away with probationers in the workhouse infirmary. The superintendent nurse lately submitted a scheme relating to the nursing arrangements, which embodied a recommendation that probationers should be dispensed with, and Dr. Hartford, the medical officer, in supporting the recommendation, stated that, on account of the smallness of the establishment, and there being no surgical work owing to the cases being mostly chronic, no satisfactory training could be given to probationers to enable them to obtain qualifying certificates at the end of three years. In these circumstances, the decision of the guardians is undoubtedly sound and we commend it to the emulation of other boards who find that similar conditions prevail. The best interests of nursing can only suffer from the continued existence, or multiplication of schools in which the training falls necessarily very far short of the proper standard. The nursing staff at Christchurch Workhouse Infirmary will in future consist of the superintendent nurse, a day charge nurse, two night charge nurses, and five assistant nurses with one year's experience. The latter are to be made "to a certain extent responsible."

### THE UNDAUNTED WORKHOUSE MASTERS.

Parliament has been scattered to the four winds of heaven without the President of the Local Government Board arriving at any decision as to the recommendations of the Departmental Committee on Workhouse Nursing. It is inconceivable that Mr. Long will listen to the demand of the Association of Workhouse Masters and Matrons that the status created by the Nursing Order of 1877 should be maintained. But when he has had time to acquaint himself with the terms of the document which this body is to present to him, we hope that he will be able to announce the course he has determined to pursue. Meanwhile, it is interesting to notice that Mr. Grayson, master of the Ipswich House, makes "an humble but sincere appeal to all masters and matrons throughout the country to show their appreciation of the earnest and undaunted efforts of those masters, who, from the beginning, have so persistently, frequently at great personal inconvenience and expense, defended their position, by promptly signing the petition." That people should be earnest and undaunted in their efforts to preserve their own authority, is neither surprising nor blameworthy. But it is a little unusual for them to glorify themselves, even in strains of humility.

### HOTEL EXPENSES FOR GRANARD NURSES.

At the meeting of the Granard Board of Guardians last week, a bill of considerable dimensions for hotel expenses for the temporary nurses at the Infirmary was presented, and elicited from some of the members the observation that this sort of thing would soon end, "as the Irish party would get the nuns back for them." But when the question of the expulsion of nuns as nurses at Granard was raised in the House of Commons, the Chief Secretary did not hold out any prospect of their return. He admitted, as all of us gladly admit, that nuns are doing great service as nurses, and that their ministrations are acceptable to the patients, in Ireland at any rate. But he told Parliament that as they do not care to perform some of the hospital duties, trained nurses are essential at Granard. The best way of getting rid of hotel expenses for temporary nurses is for the Granard Guardians to reconcile themselves to the inevitable, and conform to the regulations of the Irish Local Government Board in the future.

### NURSING AMONG THE HOP-PICKERS.

An appeal is made by the Hop-Picking Mission Committee, of which the Rev. F. G. Oliphant, Teston Rectory, Maidstone, is hon. secretary, for funds to supply a sufficient number of trained nurses to work among the different centres in Mid and West Kent during the season which commences early next month. In our issue of August 1st, we published an account by a nurse of her work among the Kentish hoppers last season, and nothing we could say would add to the strength of her practical reply to the question, "Why do hoppers want nursing?" But it may be worth while to state that the sum offered by the Hop-Picking Mission Committee to a nurse for her expenses is £5. We think, as our correspondent said three weeks ago, that a nurse who can spare the time would thoroughly enjoy the experience, and the feeling that she is of some little use to a class of people with whom she might not otherwise come in contact.

### SHORT ITEMS.

The s.s. *Menes*, which arrived at Southampton on Friday, had on board Sister H. McCurdy, of Queen Alexandra's Imperial Military Nursing Service, whose term of duty in Gibraltar has expired.—Under the will of the late Mrs. Pyne, of Royston, Herts, the Royston Nursing Association will benefit to the extent of £500.

### The Nursing Outlook.

"From magnanimity, all fear above;
From nobler recompense, above applause,
Which owes to man's short outlook all its charm."

### DEEDS OR WORDS.

THERE is a danger which the parish nurse and the school nurse alike share that they may degenerate into the women of words instead of remaining women of action. Not that the danger is peculiar to nurses, it frequently affects all those who visit the poor constantly, and who are not blessed with bigness of heart. But our concern is chiefly with the two cases mentioned, and here are specimens to explain our meaning. A parish nurse often becomes the messenger of the clergy; her bag contains not only nursing requisites but food and coal tickets, or relief in other shapes. Because she dispenses charity there is always some neighbour at hand ready to do simple tasks of cleaning or nursing work for her; and as the serious cases go to the hospital and the very poor to the infirmary the nurse is apt to feel that the personal performance of commonplace duties are not necessary, and that if she supplies the linseed one of the family can very well make the poultice. And so gradually the "nurse" as we and as probably she at first understood the term, disappears and becomes absorbed in the "district visitor," displacing deeds for kind words and small doles. The school nurse also runs the same danger of overlooking the value of personal action. Her duties also are not serious, and chiefly consist in cleansing sore heads, putting ointment to inflamed eyes, syringing discharging ears and so on. Soon the daily round and common task begins to pall, and the nurse agrees with the teacher that the mothers ought to do these little actions, and she quite forgets that it is just because the mothers have not attended and do not attend to these minor ills that the school nurses have come into existence. But the constant struggle against dirt and vermin in the poor quarters of a big city is very disheartening, and the sense of irritation and disgust grows, and sympathy disappears. Then the nurse scolds the children for being dirty, scolds the parents for letting the children be dirty, and tries to scold the teacher into excluding all dirty children. But there is no salvation by scolding; you cannot teach cleanliness by words. The duty of a nurse is to do: if she objects to this let her drop the title of nurse and call herself by some other name which signifies that her mission is not to aid, but merely to find fault.

Of course, the root of the evil in both cases results from the circumstance that single nurses are working apart from a central organisation and under no trained superintendent. A Queen's nurse working in a parish is kept up to the mark by periodical reports and professional supervision; a school nurse working from a nursing home and with an inspector criticising her work at intervals is bound to labour with her hands. And after all there is no reason why all parish nurses and all school nurses alike should not be superintended as are Queen's nurses and kept to the high level of their calling by the honour of the Jubilee Institute. In New York the nine school nurses all work under one superintendent nurse, who allots them their duties, follows them up in their rounds, and supervises all their reports and submits them to the medical officers of schools. In the London schools there are three nurses working under the School Board, and apparently with no professional supervision; and there are three or four voluntary nurses who used to be inspected by Miss Peter, of the Queen's Jubilee Institute, but who are now, in two cases at least, working entirely on their own lines. The wisest thing would be for the Board to take over all the nurses, put them all on the same footing, and place them under a nursing inspector, who would be responsible to the Board's medical officer for the manner in which the work was performed. There is no excuse, so far as the school nurses are concerned, for any slackness; for they are engaged in a great national work, in helping towards a better physique for the race of the future. They are working in State institutions, and all the religious red tape that besets the parochial nurse has no hindrance for them. Of course charity is only a crutch, and so long as the funds are dependent on the caprice of the public, or the begging powers of an honorary treasurer, the society will be more or less uncertain and unstable in its methods. There is a considerable opening for the new educational authorities to supply better hygienic methods for the scholars, including medical inspections of the schools and a crusade of cleanliness by the nurses. But it is work that is worth doing thoroughly and worth doing well; and an agency which merely sends another set of fault-finders to scold the poor for being poor, would not be new, would not be nice, would not be popular. The nurse must actually clean the dirty heads and anoint the sore eyes; she must wash the poisoned cut and dress the festering wound; she must teach by example and by practice, by sympathy and ready help. The unfortunate and neglected little children of our cities have quite enough preaching and scolding already to put up with: it does but very little good to tell them things, but let them see and they will learn with alacrity.

For the parish nurse who becomes the almoner, whose words are not so much fault-finding, but rather encouragement, we have more sympathy. Still it must be maintained that nursing is practical, a thing of deeds and not of words, and that those who fall short in acts are but poor members of the profession. It is not for nurses to deal with the great social and scientific questions which underlie poverty and disease; it is for them to help to cure the individuals who suffer.

### Lectures on Ophthalmic Nursing.

By A. S. COBBLEDICK, M.D., B.S.Lond., Senior Clinical Assistant and late House-Surgeon and Registrar to the Royal Eye Hospital.

### LECTURE XVII.—THE TREATMENT OF IRITIS. ACUTE GLAUCOMA.

*Treatment.*—This must be largely local; if any constitutional taint can be discovered, quite apart from the patient's statements, general treatment is indicated. The rest treatment applies to an inflamed iris as much as to an inflamed joint; the only way the iris can be kept at rest is by paralysing its sphincter muscle and also by paralysing the ciliary muscle, which is so constantly and unconsciously in use when we accommodate. It can be readily understood that movements of the iris, when acutely inflamed, cause great pain. Rest can be obtained and the pain much relieved by the instillation of one or two drops of gutt. atropinæ sulphatis gr. iv. ad ℥ i. three times a day; this causes dilation of the pupil and complete immobility. Another advantage of this dilation is that the free edge of the iris is drawn away from the convexity of the lens, so that if exudation is thrown out posterior adhesions (synechiæ) are not formed. A minor disadvantage is the excess of light which gains admittance through the wide pupil: this can be remedied by using a shade or a pair of dark glasses. The use of atropine must form the basis of treatment in all cases. It must also be continued until all signs of the inflammatory condition have disappeared and then gradually diminished in strength or frequency of application. If there are old signs of syphilis or a definite history of this disease, mercury in some form or other should be given; some surgeons prefer inunction whilst others find it as efficient and cleaner if given by the mouth, either as pil. hydrarg. c̄. creta or the liquor hydrarg. perchlor., to the latter of which may be added potassium iodide. If the pain is unrelieved by the action of the atropine, fomentations (poppy head) may give relief. The surgeon must also pay attention to the general health; the bowels should be regulated, fresh air, regular exercise, and a free, nourishing diet should be ordered. If there is a history of rheumatism and the iritis does not improve under atropine, the salicylates and alkalies are indicated. Sometimes the pain is not relieved by placing the iris and ciliary muscle at rest and other measures must be tried. Blisters on the temple of the affected side may be useful, but, generally speaking, they tend to aggravate the patient's condition. It is better to apply two or three leeches to the skin in the neighbourhood of the external canthus; in applying these it is well to remember that they will not take to

1/3 SCALE

FIG. 1.

skin that is *greasy* or *dirty*: it is usual to cleanse the chosen area of skin with spirit, and dry carefully; then to place the leech in a small test tube three quarters full of cotton wool and to invert the tube over the selected part. As a rule it is not necessary to interfere with them; if for any reason they need to be removed before they are satisfied, a little salt solution will make them release their hold; the leech bites should be covered with absorbent wool and treated aseptically like a surgical wound. In place of leeches the part may be cupped with Heurteloup's artificial leech (see Fig. 1); this consists of a spring knife with which to make the incision, and a series of glass cylinders fitted with air-tight pistons which can be very gradually withdrawn by means of a screw shaft.

*Acute Glaucoma.*—This is a convenient place to consider this disease; for although it is not a disease of the iris, its successful treatment depends on an operation on the iris (iridectomy). It usually occurs in middle life, and for the most part in those who suffer from hypermetropia (a condition dependent on the eyeball being too short in its antero-posterior diameter, and remedied by the use of convex glasses) especially when uncorrected by glasses. In nearly all cases the sufferer has had some serious worries—domestic or financial. The true cause of the disease has not been determined, but it is due to the normal lymph circulation *within* the eyeball—(spoken of in a previous Lecture)—becoming obstructed; the result is that although fluid continues to be formed *in* the eyeball, the channels of outlet are closed, so that the tension gradually increases and may reach such a degree as to completely spoil the sight in the eye within a few hours.

*Symptoms.*—(i.) Premonitory. These consist in short attacks of slight pain with hazy vision, lasting from a few hours to a day; halos around lights may also be complained of; another indication is impairment of accommodation, with the result that higher glasses are required for reading than the age of the patient should necessitate. In the intervals of these attacks the eye becomes normal. These symptoms may present themselves over a period of one or two years before they culminate in an acute attack. (ii.) Immediate. In the majority of cases the onset of an attack is at night time. The great pressure within the eye gives rise to a most agonising *bursting* pain, which may or may not produce *vomiting.* The pulse is quick, and in a severe case the patient may be in a state not far removed from collapse.

*Signs.*—The sclerotic is intensely and uniformly red, the pupil wide and irregularly oval, and instead of being black it has a peculiar *green* tinge; the anterior chamber is abolished, so that the iris and lens are in contact with the posterior surface of the cornea. The *tension* is very much raised, not unfrequently the eyeball feels stoney hard. Very soon the cornea becomes hazy and opacities form in the vitreous, so that it is difficult to obtain a view of the fundus. The cornea also becomes insensitive to the touch of the finger from pressure on the nerves which supply it. Most acute cases are fairly typical, and the above signs are usually more or less well marked.

### To Nurses.

WE invite contributions from any of our readers, and shall be glad to pay for "Notes on News from the Nursing World," or for articles describing nursing experiences, or dealing with any nursing question from an original point of view. The minimum payment for contributions is 5s., but we welcome interesting contributions of a column, or a page, in length. It may be added that notices of appointments, entertainments, presentations, and deaths are not paid for, but that we are always glad to receive them. All rejected manuscripts are returned in due course, and all payments for manuscripts used are made as early as possible after the beginning of each quarter.

### Queen Alexandra's Military Nursing Service for India.

### THE CONDITIONS OF APPOINTMENT.

WE have received from the India Office the revised regulations of Queen Alexandra's Military Nursing Service for India, which have just been printed:—

1. The nursing establishment consists of three grades, viz.:—

(1.) Lady superintendents.
(2.) Senior nursing sisters.
(3.) Nursing sisters.

*Note.*—The term "lady nurse" as used in the following paragraphs includes all three grades.

The numbers in these grades are subject to alteration; but at present the service is composed of 65 nursing sisters, 15 senior nursing sisters, and four lady superintendents.

2. Nursing sisters must be, at the time of appointment, over 25 and under 35 years of age, and before admission to the Service they must satisfy the Nursing Board at the India Office, in a personal interview, as to their general suitability, and must be certified by the President of the Medical Board to be physically fit for service in India. They must have had at least three years' preliminary training and service combined in a general hospital or hospitals in which adult male patients receive medical and surgical treatment, and in which a staff of nursing sisters is maintained.

3. The duration of a term of service, for all grades of lady nurses, is five years, and may be renewed for a second, and again for a third, period of five years at the option of the Government, with the consent of the lady nurse. A lady nurse who has been pronounced by a medical board to be physically fit for further service in India, and who may be specially recommended by the Commander-in-Chief in India for an extension of service, will be permitted to serve for a fourth term of five years.

4. The engagement may, however, be terminated at any time on six months' notice being given, either on the part of the Government or of the lady nurse (but see paras. 16 and 17).

### RATES OF PAY.

(In addition to free quarters, fuel, light, and punkah-pullers.)

| | | Rs. |
|---|---|---|
| 5. For a lady superintendent | ... | 300 per mensem, |
| For a senior nursing sister | ... | 200 " |
| For a nursing sister ... | ... | 175 " |

commencing from the date of embarkation for India.

A local allowance of 50 rs. per mensem is authorised for the senior lady superintendent.

An allowance of 60 rs. for the provision of uniform is authorised for every senior nursing sister and nursing sister at the end of each completed pear of service; and a grant-in-aid for the provision and maintenance of mess property is made at the rate of 50 rs. for each lady nurse on appointment and 12 rs. annually afterwards.

### LEAVE.

6. After engagement for a second, third, or fourth term of service, a lady nurse may be granted not more than one year's leave from duty, on two-thirds pay, with free passage by sea and land from and to her station. Such period will not reckon as service in any way.

7. Leave on medical certificate, either in or out of India, up to a maximum of six months during each five years' term of service, may be granted to a lady nurse by the principal medical officer of the command; during such leave, which will reckon as service, she will receive two-thirds of the salary of her grade. Such leave may be extended for a further period not exceeding six months, on the condition that the period of extension shall be reckoned as part of the one year's leave referred to in paragraph 6 on completion of a term of five years' service, should the lady nurse eventually become entitled to such leave. This extension will only be granted on the recommendation of a medical board. If the leave is taken in Europe, free passage by sea and rail is granted on the homeward, and by rail only on the outward, journey. Short leave and privilege leave may be granted at the discretion of the local authorities and as circumstances may admit.

### GRATUITIES.

8. The following gratuities on leaving the service are payable to lady nurses:—

| — | Nursing Sister. | Senior Nursing Sister. | Lady Superintendent. |
|---|---|---|---|
| For a completed term of 5 years .. .. | Rs. 500 | As Nursing Sister, with 70 rs. additional for each complete year's service as Senior Nursing Sister. | As Nursing Sister, with 70 rs. additional for each complete year's service as Senior Nursing Sister, and 140 rs. additional for each complete year's service as Lady Superintendent. |
| For a completed term of 10 years.. .. .. | 1,500 | As Nursing Sister, with 125 rs additional for each complete year's service as Senior Nursing Sister. | As Nursing Sister, with 125 rs. additional for each complete year's service as Senior Nursing Sister, and 250 rs. additional for each complete year's service as Lady Superintendent. |
| If compelled by sickness to leave India before completion of a 5 years' term, for each complete year's service .. .. | 75 | Rs. 100 | Rs. 200 |
| If compelled by sickness to leave India after completion of a first term of 5 years' service, but before completion of a second, for each complete year of the second term, and in addition to the gratuity for the completed term of 5 years as shown above .. .. | 100 | 120 | 240 |

9. No gratuity is given for service terminated by any other cause than sickness or the completion of the term of 5 or 10 years' service.

### PENSIONS.

10. Lady nurses retiring after completion of 15 or 20 years' total service will be entitled to pension at the following rates:—

| — | Nursing Sister, per annum. | Senior Nursing Sister. | Lady Superintendent. |
|---|---|---|---|
| After 15 years' service .. | £ 50 | As Nursing Sister, with £1 additional for each complete year's service in the grade of Senior Nursing Sister. | As Nursing Sister, with £1 additional for each complete years' service as Senior Nursing Sister, and £2 additional for each complete year's service as Lady Superintendent. |
| After 20 years' service .. | 60 | | |

### QUEEN ALEXANDRA'S MILITARY NURSING SERVICE FOR INDIA—*Continued.*

11. A lady nurse compelled by ill-health to retire after more than 15 but less than 20 years' total service will receive a pension of £50 a year, with an increment of £2 for each completed year over 15 years' service, and an additional £1 a year in the case of a senior nursing sister, and £2 a year in the case of a lady superintendent, for each complete year served in those grades respectively (with the addition, for a lady superintendent, of £1 a year for each complete year's service in the grade of senior nursing sister).

12. A lady nurse compelled by ill-health to retire after more than 10 but less than 15 years' service, will be granted such rate of pension, below that fixed for 15 years' service, as may be determined by the Secretary of State for India in Council on the recommendation of the Government of India.

13. If paid in India, these pensions will be payable in Indian currency at the rate of 15 rs. to the £1 sterling.

PASSAGE, ETC.

14. Passage at the public expense, subject to a deduction of 2s. a day for messing while on board ship, is granted to lady nurses when proceeding to India on appointment; and also when returning home on leave (under paragraph 6) on completion of a term of service, or if invalided home before the completion of a term, provided that they avail themselves of it within one year of the completion of their term or of the orders permitting their retirement, they are entitled to take 6 cwt. of baggage. They are also entitled to travelling expenses on the above occasions from their places of residence in England to port of embarkation, and from port of disembarkation in India to destination in that country, and *vice versa.* Claims for conveyance of baggage must be supported by vouchers. Agency and dock charges are inadmissible.

15. An outfit allowance on appointment is made at the rate of £15 for each nursing sister.

16. A lady nurse who resigns (except on account of ill-health), with less than five years' Indian service, will forthwith refund the sum of £20 in respect of her passage out (or £30 if she have failed to give the notice requiiel by Rule 4), and will not be entitled to a passage home.

17. A lady nurse who, having completed a term of service, has re-engaged and availed herself of leave to England, will be subject to the same penalties if she resigns (except owing to ill-health) before completing the term of her engagement. If, however, she has not availed herself of leave to England on re-engagement, she is subject to no penalty on resignation, except a fine of £10 if she fails to give six months' notice; and, whether she gives notice or not, she will receive free passage to England.

18. Lady nurses must be prepared to embark, if necessary, not later than 30 days from date of appointment.

### Wants and Workers.

WOULD any nurse like the weekly copies of THE HOSPITAL for the year from August 23rd, 1902. Carriage free. Apply Miss L. Weston, Harpenden Lodge, Jameson Road, Bexhill-on-Sea.

MISS K. GREG, of Lode Hill, Handforth, Manchester, offers a bicycle, in good condition and usual size, gratuitously to a district nurse to assist her in her work. She herself is getting a free wheel, and would like to feel that the machine she is giving up is useful to someone else.

### Nursing in Sierra Leone.

BY A SISTER.

HOT out there? Yes, indeed; and never cool except during the rains, when the rainfall may be as much as 40 inches in a month. I arrived in Freetown in July one year, when the rainy season was fairly well established, and I wondered if we should ever see fine weather again. This was my first visit to the tropics, and I was out there a year.

TWENTY-FOUR HOURS ON DUTY.

I was told that my duties would be principally at the Government Nursing Home for Europeans, which holds eight beds. There was one other sister in the home, and we divided the work. I found the hours were very long; one sister entered the wards at 12 noon, and stayed till the next day at noon—24 hours on duty! At night we had a boy nurse from the native hospital to sit in the wards with the patients, but, of course, Sister had to be in and out frequently all night long, or she would be certain to hear a patient calling the boy, with no response, and then on going into the ward would find him with his arms on the table, and his head reposing on them, fast asleep. This was when the patient was not very ill. If he were, the sisters did 12 hours' duty each, never going out of the ward all the time, except for a meal, when each was relieved by the other sister. It was often very trying work indeed, the heat was so intense, and not many conveniences to be had out there; but during my stay we never had more than three European patients in the home at a time.

THE NATIVE HOSPITAL.

The principal cases were malaria and blackwater fever but there were other diseases as well, and I can testify that nursing in a climate like that of Sierra Leone is a very hard task. At the same time it is a most interesting one, and the nurses' services are deeply valued. When, as occasionally happened during the dry season, there were no European patients in the nursing home, the sisters went to the native hospital and instructed the boys, holding classes for bandaging, etc., and generally superintending the work done in the six wards. Some of the boys turned out very capable nurses but, oh! how careless they were if they thought no sister would be round. They did not like it when there were no patients in the home. But the native patients in the hospital did, and smiled a welcome to us whenever they saw us appear. They knew then that things would be done more regularly; and it was nice to see sister on her rounds, and to hear her say to each in turn: "Good morning Mammy (or Daddy), how you do?" The answer would be always either "Tank God, Ma I am better," or "I lib for die, Ma" (I am going to die).

WORK DISHEARTENING.

But work there was very disheartening, as we never knew how many days we could spend at the hospital; and always just as things were going on a little better, the order would come that a European patient was being sent into the Nursing Home, and we had to say good-bye to the natives, not knowing when we should return. A European sister is badly needed as a permanency in the Native Hospital, and perhaps some day there will be one. At present the doctors have to manage with only occasional help from the sisters belonging to the Nursing Home. For many reasons I was sorry when my time was up, for it had been most interesting work, but I was glad of the rest which I needed.

### The Nurses' Bookshelf.

Practical Guide to Surgical Bandaging and Dressings. By W. Johnson Smith, F.R.C.S. (London: The Scientific Press. 167 pp. Price 2s.)

There has been quite a noticeable tendency of late to provide nurses with text-books which are to some degree behind the times. It must be pretty clear to anyone who has given much thought to the matter that this is not a proper principle at all. In her own sphere a nurse should keep herself every whit as up-to-date and *au fait* with recent advances in her art as must the medical student or practitioner. Accordingly, we are glad to see Mr. Johnson Smith's little book which, while being in accordance with modern surgical thought, is at the same time simple and concise. There are no redundancies, nor does the author enter into unnecessary speculations; all that he says is thoroughly practical and to the point. We are pleased to note the general arrangement of the chapters, the first four of which are devoted to the infection and modern treatment of wounds. In dealing with this subject first, Mr. Johnson Smith emphasises the absolute necessity that there is for a nurse, if she is to act intelligently, to have a full knowledge of the scientific principles upon which modern antiseptic methods of wound treatment are based. It is most important that this should be realised to the full, for the work of a nurse or surgical dresser, like that of the surgeon and physician may be truthfully said to lie to a great extent in assisting the sick to resist invasion by the micro-organisms of disease. The chapters which deal with splints and bandaging are profusely illustrated, and by a glance at the woodcuts it is easy to follow the descriptions in the text. It is rather to be regretted that the book is not provided with a fuller and more complete index, and perhaps this little deficiency will be corrected when a second edition of the book appears. However, it is not a sufficient matter to detract from the general excellence of the work which seems to us to fill a distinct want on behalf of both nurses and students who are commencing their work in the surgical wards of our hospitals.

Nurses and Nursing: or, How Not to Do It. By Sister Medicatrix, Ex-N.S. (Authors' and Booksellers' Co-operative Alliance, Limited, 151 Strand.) Price 6d.

This little book bears justly its sub-title, "How Not to Do It." Its instructions, for the most part, had better be ignored than obeyed. Some nursing advice there is, but only what can be got from any one of the hundreds of nursing manuals now published, and most of Sister Medicatrix's views are decidedly mischievous. For example:—"I know many instances in which the nurse has done wisely to exercise her own private judgment, and not a few where she has saved life in doing the exact opposite to what the doctor ordered." "No food is the best support." "Use no disinfectants; remember that one smell is as bad, or as good, as another." "Use no salves at all." "There is no need for quarantine." "Are eggs food? Did nature so intend them? Never, any way or in any form, administer them to a patient." Nurse Medicatrix's grammar often leaves much to be desired. But while that is bad, her opinions are worse.

Invalid Recipes. By E. E. Mann. (London: Longmans, 39 Paternoster Row. Price 2d.)

A very useful collection of recipes for tempting invalid drinks and dishes has been got together by the compiler of "Invalid Recipes." Nice little dainties are included that will appeal to delicate persons who are not actually invalids. It will be specially useful to district nurses and others, to whom a knowledge of small dishes easily prepared is invaluable in cases where the resources and means of their patients are limited.

### Everybody's Opinion.

[Correspondence on all subjects is invited, but we cannot in any way be responsible for the opinions expressed by our correspondents. No communication can be entertained if the name and address of the correspondent are not given as a guarantee of good faith, but not necessarily for publication. All correspondents should write on one side of the paper only.]

### NURSES AS BRIDESMAIDS.

Miss E. Eleanor Jarvis writes from the Grand Hotel, Eastbourne: As you have so severely, and in my opinion so unjustly, criticised the nurses' uniform worn at a wedding which took place at the West Croydon Tabernacle on July 22nd, it may interest you to know that the bride and all the bridesmaids were fully-trained nurses and members of the R.B.N.A., and therefore, I presume, had a perfect right to wear their uniform. I think, before denouncing the bridesmaids for "getting themselves up" as nurses, it would have been better if you had made sure of your fact beforehand, and not wrongly censured members of a profession which your journal professes to support. This is by no means the first time a nurse's uniform has been worn by nurses at a wedding. I read an account of a similar one in The Hospital about four or five years ago, which was admired and considered appropriate. I have a great respect for the nurses' uniform, and much objection to its abuse, being myself a trained nurse and one of the bridesmaids at the wedding on July 22nd. Surely a nurse is equally as justified in wearing uniform at a wedding as an officer of the army or navy would be. I pass over the petty innuendo that the bride's work in hospital might not have been that of a nurse (as a matter of fact she is a fully qualified member of the nursing profession), and merely conclude by expressing the opinion that an apology is due both to bride and bridesmaids.

Mr. Frederick Jayne, of "Fernlea," 3 Gonville Road, Thornton Heath, writes: Would it not have been as well to ascertain somewhat of the case referring to wedding at West Croydon Tabernacle before publishing so scurrilous a report on same—an insult to bride, bridegroom, and bridesmaids—and not satisfied with that but insult the groomsmen? I happen to be the father of the bride, and as such you will see in report of wedding, am much respected in our town. Now, first, with an ordinary mind, would any individual imagine for a moment that a doctor and qualified hospital nurse as his bride would degrade themselves or would have any cause to hunt for mock bridesmaids? The two bridesmaids were trained nurses and members of the R.B.N.A., and are naturally much disgusted by your behaviour, and I consider you have committed an insult most unjustifiable of bride, bridegroom, bridesmaids, and parents, and shall expect a full public apology in your next. I should advise you to be more careful in your remarks or you may find yourself in trouble. I sign myself as one grossly insulted. Rather singular, at the request of the bridesmaids, I sent you the report of wedding, little knowing what dangerous hands we were throwing it into.

[We deal with this subject in "Notes on News."—Ed. Hospital.]

### THE ONE-YEAR NURSE.

Mrs. Richmond, matron of Luton Workhouse, writes: May I again trouble you for the use of your columns for the few following remarks in reply to "A Workhouse Infirmary Nurse"? It is to be regretted that there are so many nurses who, when any point crops up which does not meet with their approval, instead of bringing something tangible against it which all fair-minded people would gladly consider, write a letter consisting mainly of abuse and hide themselves behind a *nom de plume*. Whatever qualifications they may have, high principles and moral courage cannot be attributed to them. Your correspondent "A Workhouse Infirmary Nurse" appears to belong to this order. She certainly is more proficient in imputing base motives to masters and matrons than in producing sound arguments against the question at issue. Nurses should remember when attempting to damage the reputation of others, that they have not by any means a perfectly clean slate, and judging from verbal and written matter which comes to hand from time to time, neither the size of the slate nor the blemishes upon it are diminishing. Nobility of personal character as well as dignity and status of professional position might well be included in the education of nurses. Two facts have, I think, escaped her notice: firstly, that the statistics *re* trained matrons are Mr. Davy's, Local Government Board Inspector, and that it is his word which she is doubting; secondly, that the way this recommendation of the Departmental Committee has been dealt with illustrates how some people will suppress and distort important information to gain their own ends. The fallacy of your correspondent's concluding remarks is self-evident to anyone conversant with both sides of the question, and needs no comment from me.

### NUNS AS NURSES.

"Cecilia" writes: You recently paid an extremely high tribute to the nuns for the services they have rendered in times past, but it is curious that you complain of nuns as nurses because they have not a sufficiently high religious tone. Exception may be taken to them professionally, one may object to their dress, their rules, their lack of a secular certificate; but to complain "that they have not yet learned as a simple rule 'All for Thy sake Lord'" when they are the only body of nurses to whom this is the very keynote of existence seems rather beside the mark. And as professional nurses are "ousting nuns from the highest form of charity," the reason given being that nuns have "failed to do all for our Lord's sake," are we to conclude that nurses succeed because they work all for His sake? I have known and worked with nurses of all classes and all creeds, and I am not alone in saying that nurses are one of the most irreligious bodies of women. The nursing profession has ceased to have any connection with religion, not only in theory, but too often in personal practice. The object of most nurses is to earn a living, and a very laudable object, but there is no occasion to cover it with cant and imply that they do the "lowest and most humble deeds" because of a "Christlike" spirit which many do not even profess. Let nurses and nursing nuns be criticised on the same ground, *i.e.* professionally, and to any extent; but not religiously, for this alienates the sympathy of every Catholic. Within the last few months two friends of mine have required skilled nursing. One was a Catholic with diphtheria, who had two nuns to nurse her, and I heard of nothing but love and gratitude between them and their patient. The other was a Protestant with pneumonia. She sent for a nurse on the doctor's recommendation from the private staff of a large hospital. When she had been sent out of the house by her patient's husband after a fortnight of continual unpleasantness, my friend said to me, "I will never have another trained nurse in my house again as long as I live." I always try and defend my profession to the outside world by saying that all nurses are not like such an unfortunate specimen, &c., &c., but when such experiences multiply around one it becomes more difficult. There is no doubt that whatever nurses may still be when in hospital, they are not nearly as popular in private work as they are supposed to be by those whose ideas seem summed up in professionalism.

### Presentations.

Derbyshire Royal Infirmary.—Miss Wilkinson, late matron of the Royal Infirmary, Derby, before leaving was presented with a gold bracelet and gold brooch from the nursing staff. The resident staff and others presented her with silver-backed brushes and other toilet articles mounted in silver.

Dewsbury Infirmary.—Miss E. Nunn, on leaving the post of matron of Dewsbury and District General Infirmary, after nine years' devoted service, in order to be married, was presented last week with a gold and diamond brooch by the Board; with a case containing a silver-mounted hair-brush, mirror, and comb by the nursing, dispensing, and domestic staff; and with a gold bracelet by the medical staff.

### Appointments.

Axminster Cottage Hospital.—Miss Rosa M. Ford has been appointed sister. She was trained at Guest Hospital, Dudley, and has since been staff nurse at the West London Hospital, Hammersmith.

Bramley Union Infirmary.—Miss Mary A. Ray has been appointed superintendent nurse. She was trained at Sunderland Union Infirmary, and has since been charge nurse at South Shields Infirmary, and charge nurse and midwife at Keighley Union Infirmary. She holds the L.O.S. certificate.

Brandon Infectious Diseases Hospital.—Miss Ellen Maude Cadby has been appointed nurse in charge. She was trained at Barrow-in-Furness Hospital, and has since been charge nurse at Darlington Fever Hospital and at Harrogate Fever Hospital.

City Fever Hospital, Birmingham.—Miss Evelyn Frost has been appointed head nurse. She was trained at Brownlow Hill Infirmary, Liverpool, and has since been charge nurse at the Fountain Hospital, Tooting, London, and sister of the Army Nursing Reserve at Netley, Portsmouth, and Winchester Military Hospitals.

Dewsbury General Infirmary.—Miss E. H. Grime has been appointed matron. She was trained at Manchester Royal Infirmary, where she was afterwards staff nurse. She has since been head nurse at the General Infirmary, Hertford, and assistant matron at Taunton and Somerset Hospital.

Harton Hospital, South Shields.—Miss E. Wigley has been appointed charge nurse. She was trained at Firvale Hospital, Sheffield, where she was afterwards sister. She has since been sister at the Union Infirmary, Swansea, South Wales.

Incorporations Infirmary, Shirley Warren, Southampton.—Miss Jessie F. Ballantyne has been appointed matron. She was trained at the Cottage Hospital, High Barnet, and Guy's Hospital, London, and has since been sister, night superintendent, and assistant matron at Lewisham Infirmary.

Ipswich Workhouse Infirmary.—Miss Amelia Coleman and Miss Constance Frances Ogilvie have been appointed charge night nurses. They were both trained at Woolwich Union Infirmary. Miss Coleman has also been nurse at Berks County Asylum, and first assistant nurse at the North-Eastern Fever Hospital, Tottenham. Miss Ogilvie has been staff nurse at the North-Eastern Fever Hospital, Tottenham.

Leeds Union Infirmary.—Miss Mabel Ethel Maughan has been appointed charge nurse. She was trained at the Mile End Infirmary, London, where she has also been staff nurse. She has since been engaged in private nursing.

North-Eastern Hospital for Children, Hackney Road, N.E.—Miss F. M. Scott Cavell has been appointed assistant matron. She was trained at St. John's House, London, and has since been assistant matron at the Hospital for Women, Liverpool.

Queen Alexandra's Royal Naval Nursing Service.—Miss M. M. Haslock has been appointed sister. She was trained at the Seamen's Hospital, Greenwich, and has since been night sister at Walsall and District Hospital. She holds the L.O.S. certificate.

Royal Victoria Hospital, Belfast.—Miss A. Hartley and Miss L. Brabazon have been appointed staff nurses. They were both trained at Sir Patrick Dun's Hospital, Dublin, and have since been on the private nursing staff.

### Echoes from the Outside World.

### Movements of Royalty.

The King has been at Marienbad since the end of last week, drinking the waters. The first morning after his arrival he had an unpleasant experience. Having had one glass of water in his hotel at half-past six, the King went to the spring at seven, accompanied by Sir Stanley Clarke. After receiving his portion he commenced to walk, according to regulations, glass in hand, for a few minutes, followed by some people who recognised him, and before long several hundreds were running after him, whilst others rushed in front to get a better view. With the assistance of the police he reached a bench and sat down, only to find another dense circle form round him at once. His Majesty looked annoyed, and remarked afterwards in German, as he shook hands, at the door of her shop, with Fraulein Pistl, the daughter of the hatter, whom he remembered seeing on a previous occasion, "Why do the people run after me like this; it is very disagreeable? How stupid some people are!" Since then the Burgomaster of Marienbad has issued an urgent request to all visitors not to molest his Majesty, and it appears to have had the desired effect. The King leaves for Vienna on the 31st inst.

Queen Alexandra arrived at Balmoral Castle on Saturday last. Previous to her arrival, the children of the Prince and Princess of Wales had arrived from Abergeldie Castle to welcome her. The Balmoral clansmen, in full dress, were drawn up at the entrance to the Castle, and gave three ringing cheers. The Queen is accompanied by Princess Victoria.

### The Prorogation of Parliament.

Parliament was prorogued on Friday, and the King's Speech was read by the Lord Chancellor. Reference was made in the document to the various measures which were passed during the session, and in the course of the Speech, his Majesty said that he was glad to have been able, within the last few months, to visit his people both in Scotland and Ireland, and that the warm expressions of good will with which he was everywhere received had greatly touched him. In Ireland his visit to the capital, to Belfast, the chief centre of industrial enterprise, to Londonderry, through Connemara to Galway, and to Cork, enabled him to realise how much was being attempted, and by how many agencies, to improve the housing accommodation of the working population, to stimulate commercial activity, to advance the methods of agriculture, to develop technical education, and to provide for the sick and infirm. Much remained to be done, but it was with feelings of the deepest gratification that the King noticed signs of increasing concord between all classes in Ireland, presaging, as he hoped, a new era of the united efforts for the general welfare.

### Memorial to the Empress Frederick.

In the English church at Homburg—where she frequently attended service—a memorial tablet for the late Empress Frederick was unveiled last week. It is the work of Mr. W. Ohly, and takes the form of four reliefs placed in the spandrils of the arches in the aisle, representing the four evangelists, and it has a suitable inscription beneath it. Princess Frederick Charles of Hesse, with her four eldest sons, and the Duke of Cambridge were present, Sir Frank Lascelles, the British Ambassador, representing the King, who, he said, had taken a great interest in the matter and much regretted that he was unable to be at the ceremony. As, however, that was impossible, his Majesty had commanded the Ambassador to act in his name. His Excellency then removed the British flag which had up to that time veiled the portrait of the late Empress Frederick, also uncovering the reliefs at the same time. Canon Teignmouth Shore, in a short speech, alluded to the fact tha the late Empress had attended divine service in that church the first time after the crushing sorrow of her illustrious husband's death.

### Trouble in the Balkans.

Owing to trouble in the Balkans, Russia is said to have despatched a Black Sea fleet. As yet its destination is unknown, but the squadron is ordered to sail for Turkish waters. The reason for this move is that the Czar is smarting under a peculiarly grievous injury. When the first Russian consul was killed at Mitrovitza he abstained from pressing for the death sentence on the ground that the murderer was an Albanian. Now a second consul has been murdered, and the Czar has naturally demanded the punishment of the offenders. The Sultan and the Porte tendered an apology, but this the Czar did not consider sufficient. The Sultan has since promised every satisfaction, and the gendarme with an accomplice who murdered the consul have already paid the death penalty; this has caused great anger among the lower classes at Monastir, where the execution took place, and wholesale massacres of the Bulgarian population are said to have taken place. Severe fighting is reported in different parts, and altogether the country is in a most disturbed condition. There is revolution in all directions, and the principal object of Russia in sending a squadron is, Count Lamsdorf says, to maintain peace in the Balkans. In consequence of the insurrectionary movement it is stated that the Turkish Government are calling out 52 battalions from the European provinces of Turkey.

### Cyclone in Jamaica.

Last week the unfortunate inhabitants of the island of Jamaica were visited by a terrific cyclone. Only 50 lives were lost, but the damage done to the plantations in the east and north-east, where the cyclone struck with full fury, was terrific. Buildings of all kinds, both public and private, were destroyed, and many of the fruit plantations were entirely swept away. The loss at the lowest computation is a million sterling, and the banana plantations—the growing of this fruit is now the staple industry of Jamaica as sugar was till ten years ago—have had their entire crop devastated. With good work, care, and expenditure, the banana plant will yield again in twelve months, but not before. Meanwhile, many of the firms have suffered severely financially, and thousands of persons are rendered homeless. Prompt steps are being taken to relieve the suffering. The King has sent a message expressing his deep regret for the calamity, and his warm sympathy with the victims. Sir Alfred Jones has offered to contribute £250, and also to provide for free conveyance of stores to relieve the distressed; the West India Committee have appealed for subscriptions, and the Archbishop of the West Indies, who is now in England, has also written a letter on the effect of the disaster upon the industries and prosperity of the island.

### The Humbert Trial.

The hearing of evidence in the Humbert trial was continued last week, and brought to a close on Monday. Counsel for the accused elicited some rather damaging disclosures respecting irregularities which had been committed by officials in the preparation of the case for the prosecution. Madame Humbert made two long statements in the course of the proceedings, with the view of explaining certain obscure points in her case. She also renewed her promises of complete revelation after counsel had addressed the Court. The Public Prosecutor in his speech asserted that the Crawfords and their millions were alike mythical, and were a pure invention of Madame Humbert.

### For Reading to the Sick.

### THE LIGHT OF HOPE.

I STOOD beside yon fountain, where the sun
Looked on the waters as they rose and fell
Through the calm air unceasingly, with plash
Monotonous. Their column only gave
Back to the eye a glimmer cold and pale.
Sudden, a wind descending smote the trees
That stood around, and smote the waters too
As they sprang upward; marring, as it seemed,
The fair proportions of their pillared height:
But as the breeze seized thus upon the jet
And broke it into spray, a thousand gems
Flashed in the sunshine, and the water-cloud
Gave forth a Rainbow, radiant as the first
Set by our Father as His sign in Heaven.

O tossed with tempests and not comforted!
O tried and smitten one! thy weary heart
Must read its lesson here. Thy Saviour's love
(Shaken and broken though thy spirit be)
Sends down this visiting of stormy grief
To mark thee with His Bow of Promise now,
And keep thee for His own eternally.

*T. V. Fosberry.*

Let us then think only of the present, and not even permit our minds to wander with curiosity into the future. This future is not yet ours; perhaps it never will be. It is exposing ourselves to temptation to wish to anticipate God, and to prepare ourselves for things which He may not destine for us. If such things should come to pass, He will give us light and strength according to the need. Why should we desire to meet difficulties prematurely, when we have neither strength nor light as yet provided for them? Let us give heed to the present, whose duties are pressing; it is fidelity to the present which prepares us for fidelity in the future.—*Fénelon.*

If from the shores of eternity we cast back our gaze over the path we have travelled in this world, which regions will shine most brightly and beautifully in the view? Not, I think, those that have seemed to be joyous in the passing—not the years of youth and health and strength and earthly happiness—but much rather the spaces that here have seemed perhaps the darkest and dreariest; for these have drawn us nearer to God, these have been fullest of prayer, on these have fallen the purest brightest rays from the Father of lights, and from Him who is the brightness of that Father's glory and the Light of the World.—*Bishop Walsham How.*

Learn of Jesus to finish all that God gives you to do. Strive for completeness in all your life, your work. Leave nothing undone that comes clearly to be done. Do it to the very best of your power, whatever it may be. So may God grant that when our earthly life comes to its close we may be able to commend our souls to Him, through Jesus Christ our Lord.—*R. W. Randall.*

One there lives whose guardian eye
Guides our earthly destiny;
One there lives, who, Lord of all,
Keeps His children lest they fall;
Pass we, then, in love and praise,
Trusting Him through all our days,
Free from doubt and faithless sorrow,—
God provideth for the morrow.

*R. Heber.*

### Notes and Queries.

FOR REGULATIONS SEE PAGE 257.

*Apoplexy.*

(220) Will you kindly tell me what to do for an apoplectic patient while awaiting the arrival of a doctor?—*Sparrow.*

As apoplexy is due to hemorrhage in the brain the main thing is to keep the patient as undisturbed as possible so that the bleeding may stop. Loosen the clothes round the neck and chest, and *slightly* raise the patient's head. Apply cold water to the head, or an ice bag if ice is to be obtained. Be careful there is no obstruction to the breathing; if there be the lips should be separated and the lower jaw pressed forward, and if necessary the tongue must be pulled forward too. Let the doctor superintend the removal of the patient to bed.

*Monthly Nurse.*

(221) I was engaged to be quite free to nurse a lady in her confinement on July 13th and kept waiting three weeks. Can I demand my fee from July 13th?—*B.*

If you were engaged for July 13th your month should begin from that date, but when engaging to nurse a lady you should always arrange in writing what you expect to receive if your services are not required at the time expected, or not at all.

*Dispensing.*

(222) Will you kindly tell me of a college where a lady could study for the minor Pharmaceutical Examination? I may add that she already holds the certificate of the Apothecaries' Hall and has done three and a half years' practical work in a dispensary.—*M. C.*

Write and ask the Secretary, the Pharmaceutical Society, 17 Bloomsbury Square, W.C.

*Preliminary Training.*

(223) Please inform me what to learn and what books to study, as I am desirous of becoming a nurse in a year's time.—*E. A. P.*

Obtain a simple book on Physiology, Anatomy, and Bandaging. Learn also cooking, household management, and bookkeeping, which are essential to the higher branches of nursing.

*Hospital Training.*

(224) Will you kindly tell me at which of the London fever hospitals it is possible to begin training at the age of 18?—*B. M. H.*

No hospital of high standing accepts such young probationers. The strain of nursing at so early an age incapacitates most girls for the work, and militates against their future success.

I am a ward maid and wish to become a nurse, will you please inform me of an infirmary where they are wanting probationers, and give particulars of the salary, uniform, etc.?—*L. G.*

If you look in our advertisement columns you will probably find some to suit you.

*Home.*

(225) Can you tell me of a home near London where an elderly epileptic lady would be taken at a moderate charge?—*X. Y. Z.*

The National Society for the Employment of Epileptics, 12 Buckingham Street, Strand, W.C., might help you.

*Etiquette.*

(226) When patients have a disagreement with their doctor and another practitioner is called in, is it usual for the nurse to resign in consequence?—*Nurse G.*

Certainly not.

*Seaside Homes.*

(227) Will you kindly inform me where I can obtain a list of seaside convalescent homes?—*A. M. T.*

There is a full list of convalescent homes in "Burdett's Hospitals and Charities," published by the Scientific Press.

**Standard Nursing Manuals.**

"The Nursing Profession: How and Where to Train." 2s. net; 2s. 4d. post free.

"Nursing: Its Theory and Practice." (Revised Edition). 3s. 6d. post free.

"Surgical Ward Work and Nursing." (Revised Edition). 3s. 6d. net.; 3s. 10d. post free.

"Practical Handbook of Midwifery." (New Edition). 6s. net; 6s. 3d. post free.

"Notes on Pharmacy and Dispensing for Nurses." 1s. post free.

"Fevers and Infectious Diseases." 1s. post free.

"The Art of Massage." (New Edition). 6s. post free.

## Figures and Tables

**Fig. 1. f1:**